# Residues Essential for Panton-Valentine Leukocidin S Component Binding to Its Cell Receptor Suggest Both Plasticity and Adaptability in Its Interaction Surface

**DOI:** 10.1371/journal.pone.0092094

**Published:** 2014-03-18

**Authors:** Benoit-Joseph Laventie, Frédéric Guérin, Lionel Mourey, Mira Y. Tawk, Emmanuel Jover, Laurent Maveyraud, Gilles Prévost

**Affiliations:** 1 Université de Strasbourg-CHRU, Fédération de Médecine Translationnelle de Strasbourg, EA 7290 Virulence bactérienne précoce, Institut de Bactériologie, Strasbourg, France; 2 Institut de Pharmacologie et Biologie Structurale (IPBS), Centre National de la Recherche Scientifique (CNRS), Toulouse, France; 3 Université de Toulouse, Université Paul Sabatier, IPBS, Toulouse, France; University of California, San Francisco, United States of America

## Abstract

Panton-Valentine leukocidin (PVL), a bicomponent staphylococcal leukotoxin, is involved in the poor prognosis of necrotizing pneumonia. The present study aimed to elucidate the binding mechanism of PVL and in particular its cell-binding domain. The class S component of PVL, LukS-PV, is known to ensure cell targeting and exhibits the highest affinity for the neutrophil membrane (*K_d_*∼10^−10^ M) compared to the class F component of PVL, LukF-PV (*K_d_*∼10^−9^ M). Alanine scanning mutagenesis was used to identify the residues involved in LukS-PV binding to the neutrophil surface. Nineteen single alanine mutations were performed in the *rim* domain previously described as implicated in cell membrane interactions. Positions were chosen in order to replace polar or exposed charged residues and according to conservation between leukotoxin class S components. Characterization studies enabled to identify a cluster of residues essential for LukS-PV binding, localized on two loops of the *rim* domain. The mutations R73A, Y184A, T244A, H245A and Y250A led to dramatically reduced binding affinities for both human leukocytes and undifferentiated U937 cells expressing the C5a receptor. The three-dimensional structure of five of the mutants was determined using X-ray crystallography. Structure analysis identified residues Y184 and Y250 as crucial in providing structural flexibility in the receptor-binding domain of LukS-PV.

## Introduction

*Staphylococcus aureus* largely relies on the secretion of toxins and other virulence factors such as superantigens and proteases for its virulence, targeting various actors of innate immunity [1], [2]. Staphylococcal leukotoxins, a subfamily of pore-forming toxins, appear to simultaneously confer to *S. aureus* high virulence and protection against the host's immune system. With the exception of α-hemolysin, which is homo-heptameric [3], leukotoxins are bipartite toxins, formed by the non-covalent association of two distinct proteins, a class S and a class F component of approximately 31 and 34 kDa, respectively, into a likely octameric species [4], [5], [6], [7]. To date, 7 bipartite leukotoxins have been identified in *S. aureus*: Panton-Valentine leukocidin [8], LukM/LukF'-PV [9], two γ-hemolysins [10], [11], LukE/LukD [12] and a variant thereof [13], and more recently, LukH/LukG [14] also named LukAB [15]. *S. intermedius* has also been shown to express a LukS-I/LukF-I leukotoxin, [16], while certain related genes can be found in other *Staphylococcus* species.

Four of these leukotoxins are involved in human pathogenicity. Panton-Valentine leukocidin (PVL) is associated with necrotizing skin infections, such as boils [17], [18], and plays an important role in the poor prognosis of necrotizing pneumonia [19], [20], [21]. While the two γ-haemolysins HlgA/HlgB and HlgC/HlgB are not associated with a specific disease, they are nonetheless expressed by over 99% of *S. aureus* strains [22], [23] and are thought to increase the severity of the infection [24], [25]. LukE/LukD has been reported as a dermonecrotic toxin and involved in bullous impetigo [26]. The toxic action of leukotoxins results from a complex mechanism which has been described in the case of HlgA/HlgB [7], [27], [28], [29] and is characterized by: (i) binding of the S class component on the target cell membrane, which requires the presence of a specific receptor, (ii) recruitment of the F class component, (iii) dimerization possibly accompanied by conformational rearrangement, (iv) formation of an octameric prepore, and (v) pore formation across the membrane. During this process, both class S and F proteins are faced with a dual environment: a hydrophilic milieu, when secreted by the bacteria upon infection, and a hydrophobic milieu, when forming the pore in the membrane. Independently of pore formation, leukotoxins are able to rapidly activate cellular signalization [30], [31], including an increase in intracellular calcium concentration and chemokine secretion [32].

**Figure 1:**
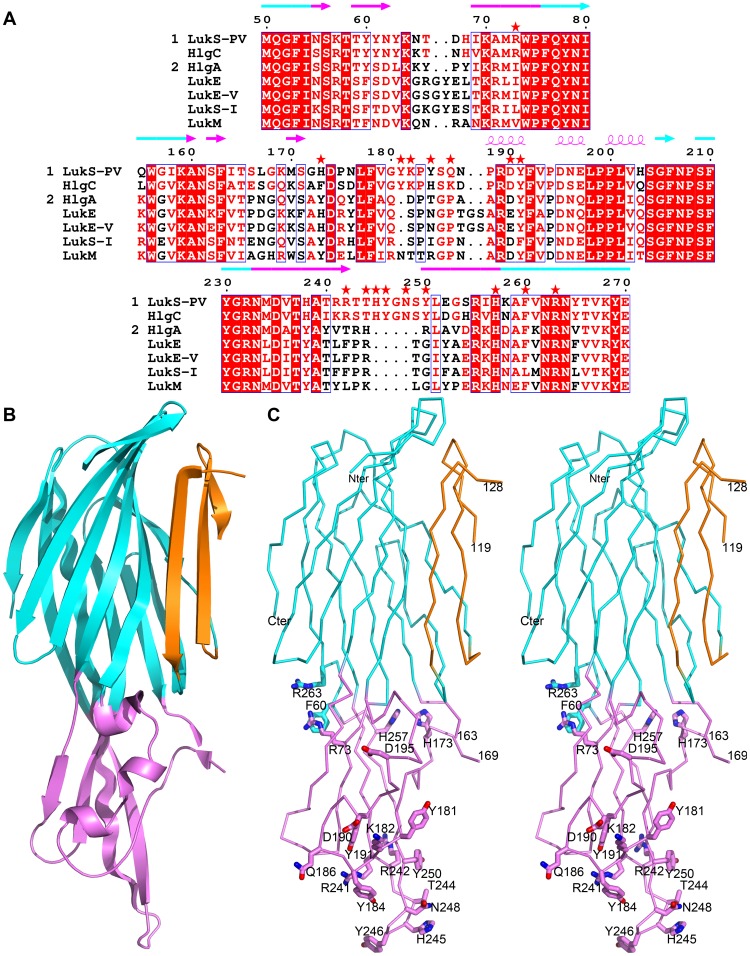
Positions selected for mutations in LukS-PV. A. Sequence alignment of the three stretches of residues constituting the rim domain of class S components of leukotoxins. Numbering corresponds to the mature LukS-PV protein. Red asterisks indicate positions selected for mutation. Strictly conserved residues are indicated on a red background, while similar residues in group 1 (LukS-PV and HlgC) or in group 2 (all others) are indicated with red letters. The secondary structure of LukS-PV is indicated above the alignment, colored according to the corresponding structural domain: β-sandwich (cyan) and rim (purple). GenBank accession numbers are LukS-PV: CAA51251.1, HlgC: AAA26638.1, HlgA: AAA26637.1, LukE: CAA73667.1, LukE-V: BAB47174.1, LukS-I: CAA55782.1, LukM: BAA97866.1. B. Schematic representation of the three dimensional structure of LukS-PV [35], PDB entry 1T5R, highlighting the three structural domains: β-sandwich (cyan), stem (orange) and rim (purple). C. Stereo view of the Cα trace of wild-type LukS-PV. Residues selected for mutations are displayed as sticks and labeled.

At the molecular level, sequence identity varies from 55 to 79% within a given class, when excluding LukH (LukA) or LukG (LukB). When these proteins are included in the comparison, sequence identities drop to about 30–34% [14]. Similarity across classes remains below 30%. The three-dimensional structure of the soluble forms of several leukotoxin components are known [33], [34], [35], [36] and display a similar fold, organized around a central domain formed by two six-stranded antiparallel β-sheets (Fig. 1). This so-called *cap* or *core β-sandwich* domain is the most conserved region, and is the location where most protein-protein interactions found in the pre-pore and pore occur. Two additional structural domains are also found: the *rim* domain anchors the protein to the membrane surface [7], [37], [38], [39], while the *stem* domain, closely apposed to the *core β-sandwich* in the soluble form, contributes two β-strands to the pore β-barrel. Interestingly, the *rim* domain is the least conserved domain, possibly resulting in variable cell specificities, depending on the leukotoxin involved.

PVL displays a narrow cellular spectrum, restricted to human and rabbit polymorphonuclear neutrophil leukocytes, PMNs, monocytes, and macrophages [31]. The binding of LukS-PV has been shown to be a prerequisite for LukF-PV binding and the subsequent activation of PMNs. Native or recombinant LukS-PV displays a *K*_d_ as low as 0.07 nM on neutrophils and 0.02 nM on monocytes [31] which is the highest known affinity for leukotoxins. Since the binding of LukS-PV to the membrane is a saturable process, the necessary presence of a LukS-PV receptor on the cell surface was thus proposed [31], [40]. This was recently confirmed by Spaan *et al*. who showed that the C5a receptor is required for the binding of LukS-PV to human neutrophils [41]. This receptor is also likely involved in the binding of HlgC, since both LukS-PV and HlgC were shown to compete for a common receptor [31]. In the case of LukE, receptors CCR5, CXCR1 and CXCR2 have been identified as binding partners on the cell surface [42], [43], whereas LukH/LukG (LukA/LukB) binds to the CD11b subunit of the integrin Mac-1 [44].

In order to identify the LukS-PV residues involved in binding to the C5a receptor at the cell surface, an alanine scanning site-directed mutagenesis strategy was adopted. Indeed, mutation into alanine is considered to allow good conservation of molecular structures, whereas the removal of polar or charged residues often alters the interacting capacity of proteins. Charged or polar residues from the *rim* domain or residues that are conserved in LukS-PV and HlgC, but not in S component of other leukotoxins, were more specifically targeted. Nineteen residues were selected and investigated for their ability to bind to neutrophils as well as activate the latter and form a functional pore. Five of the most impaired mutants were crystallized and their three-dimensional structure determined.

## Results

### Selection of mutated positions

LukS-PV and HlgC were previously shown to display similar cellular spectra and to compete for a common membrane site when binding to cells [31]. Moreover, the *rim* domain was shown to likely interact with the cell membrane within the pore [7], [45]. Therefore, the sequences of the *rim* domains of class S components were compared, in order to identify conserved positions in LukS-PV and HlgC, but not in other class S proteins (Fig. 1A). The *rim* domain encompasses three stretches of residues: S56–F76, A160–S204 and M234–R263, with the least conserved regions being Y60–R73, F163–E197 and T240–V261, corresponding to loops in the LukS-PV structure (Fig. 1B, C). Positions corresponding to polar or charged residues, with their side-chain oriented towards the exterior of the protein (as depicted on the structure of wild-type LukS-PV (PDBID 1T5R), Fig. 1C), were thus selected for mutation as these residues are more likely to be involved in interactions either with the target cell membrane or with a receptor at the cell surface.

### Production, purification and characterization of LukS-PV mutants

With the exception of the D195A and R241A mutants, all proteins were produced and purified to homogeneity, as assessed by Coomassie-blue stained SDS-PAGE. For the D195A and R241A mutants, production yields were too low to allow for purification, likely due to mutation-induced aggregation of the fusion protein.

### Binding capacities of LukS-PV mutants

**Figure 2:**
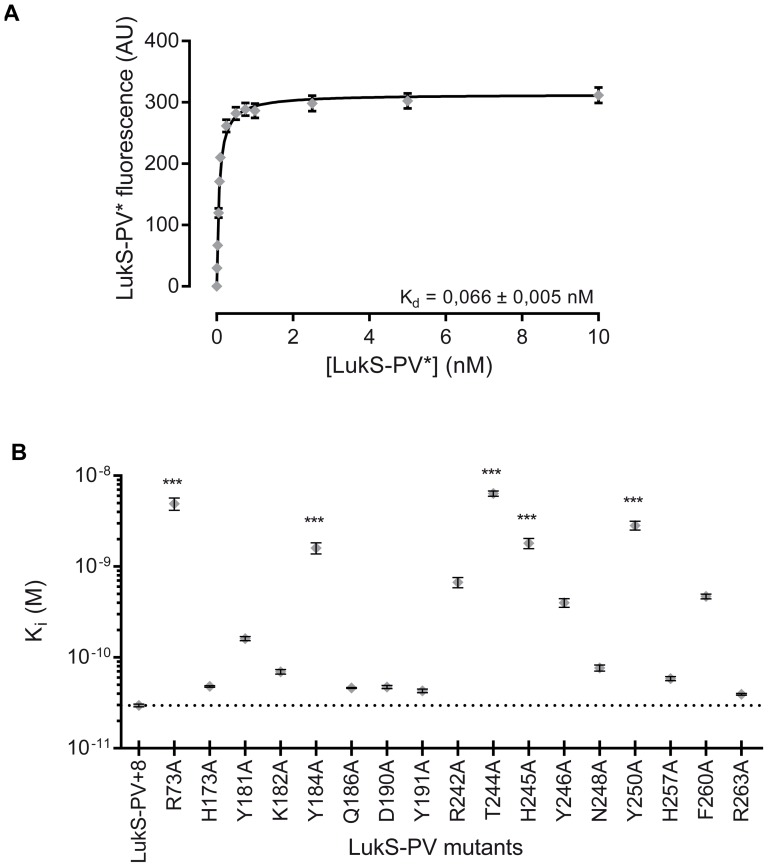
Binding properties of LukS-PV* and LukS-PV mutants to hPMNs and U937-C5aR cells. A. Flow cytometry measurement of LukS-PV* and fluorescein-labeled LukS-PV G10C binding to human PMNs and U937-C5aR cells (*n* = 3). B. Graphic representation of the *K*_i_ values obtained for wild-type or mutant LukS-PV. The dotted line corresponds to the value of wild-type LukS-PV. Error bars represent the 95% confidence interval. Statistical analysis: **: *p*<0.01, ***: *p*<0.001 (*n* = 3).

Binding capacities of wild-type or mutant LukS-PV to hPMNs were evaluated by competition experiments with a functional fluorescein-labeled LukS-PV G10C mutant (LukS-PV*). The *K_d_* of LukS-PV* for hPMNs was measured at 0.066±0.005 nM using flow cytometry (Fig. 2A), which is in good agreement with previously published data [31]. The concentration of wild-type or LukS-PV mutants required for 50% inhibition of labeled competitor binding (EC_50_) was calculated, allowing to derive *K_i_* values (Fig. 2B and Table 1).

**Table 1:** Binding properties as well as cellular and pore-forming activities of wild-type and LukS-PV mutants^1^.

Mutation	*K_i_±*95% (pM) ± confidence interval	Relative affinity to LukS-PV	Ca^2+^ entry slope (% of max/s) ± SEM	Ethidium entry (30 min) (% of control) ± SEM
**LukS-PV**	26±1.1	-	1.4±0.05	100
	(120±23)	-		
**T244A** 2	6,190±560	238	0.50±0.093	25±5.9
	(11,000±2,000)	(92)		
**R73A** 2	5,080±1,000	195	0.66±0.065	41±11
	(12,000±5,200)	(100)		
**Y250A** 2	2,970±370	114	0.77±0.17	46±14
	(5,700±680)	(48)		
**Y184A** 2	1,800±110	69	0.31±0.056	11±4.3
	(4,100±470)	(34)		
**H245A** 2	1,800±260	69	1.1±0.059	80±6.7
	(4,200±900)	(35)		
**R242A**	619±92	24	1.2±0.076	83±4.3
**F260A**	427±30	16	1.2±0.11	88±2.2
**Y246A**	365±47	14	1.0±0.098	80±6.7
**Y181A**	155±12	6	0.95± 0.045	54±5.2
**N248A**	74±8.2	2.8	0.93±0.078	77±7.6
**K182A**	68±5.2	2.6	1.0±0.062	79±3.4
**H257A**	56±4.5	2.2	1.3±0.12	96±5.5
**H173A**	45±1.3	1.7	1.2±0.073	73±5.8
**D190A**	44±2.6	1.7	1.3±0.04	90±3
**Q186A**	43±0.67	1.7	1.1±0.072	80±4.4
**Y191A**	41±2.9	1.6	1.9±0.16	111±2.4
**R263A**	38±1.3	1.5	1.4±0.068	90±2.8

1 *K_i_* values, calcium entry slope and ethidium entry at 30 min were obtained for wild-type LukS-PV and all mutants with hPMNs. *K_i_* values with U937-C5aR cells were obtained for wild-type LukS-PV and for the most affected mutants (values given in parenthesis).

2 Mutations causing a significant decrease in LukS-PV affinity for hPMNs (*p*<0.001, one-way ANOVA with Dunnett's post test).

Among the 17 tested mutations, twelve did not significantly alter LukS-binding to hPMNs, with the corresponding proteins displaying a binding capacity only affected by a factor smaller than 25-fold. The mutants exhibiting significantly decreased binding abilities with respect to wild-type LukS-PV were: R73A, Y184A, T244A, H245A, and Y250A, with *K_i_* values ranging from 1.8 to 6.2 nM, *i.e.* a 69- to 238-fold increase compared to the *K_i_* value of wild-type LukS-PV. No single mutation was found to completely prevent binding of LukS-PV (Table 1).

Undifferentiated U937 cells were mostly insensitive to the binding of LukS-PV, for concentrations of LukS-PV* up to 500 nM, yielding a calculated *K_d_*>20 μM. By contrast, U937 cells expressing C5aR, the LukS-PV receptor, bound LukS-PV* with a *K_d_* of 0.32 nM, thus 5-fold higher than for hPMNs. Furthermore, the five LukS-PV mutants R73A, Y184A, T244A, H245A and Y250A were strongly affected in their binding to U937-C5aR with calculated *K_i_* ranging from 4 to 12 nM, compared to 1.12 nM for wild-type LukS-PV, thus confirming comparable influences of these mutations to those tested with hPMNs.

### Ability of LukS-PV mutants to activate neutrophils

**Figure 3:**
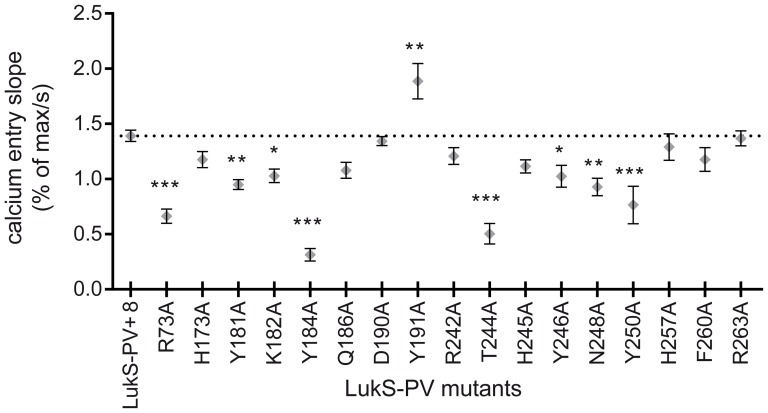
Biological activity of LukS-PV and corresponding mutants: rise of cytoplasmic calcium concentration due to human neutrophil activation. Values represent the calcium entry slope expressed in percent of maximum calcium fluorescence (after neutrophil lysis with Triton X-100 0.05% v/v) per second. Statistical analysis: ns, non-significant; *, *p*<0.05; **, *p*<0.01; ***, *p*<0.001 (one-way ANOVA with Dunnett's post test, *n* = 6).

Activation of Fluo3-loaded hPMNs was evaluated by the ability of LukS-PV mutants to induce variations in intracellular calcium concentration [40], [46]. A limiting concentration of wild-type LukS-PV or mutants was used (0.02 nM) with an excess of LukF-PV (10 nM). This concentration of LukF-PV was chosen as it is closed to the reported *K_d_* value for the binding of LukF-PV to LukS-PV [47] Wild-type LukS-PV and mutants led to an almost immediate increase in intracellular calcium concentration. Only four mutants, R73A, Y184A, T244A and Y250A, displayed an increased lag-time prior to calcium entry and a significantly reduced calcium entry slope, *i.e.* less than 55% of the LukS-PV control (*p*<0.001, one-way ANOVA) (Fig. 3 and Table 1). LukS-PV mutants Y181A, K182A, Y246A and N248A also had a significantly decreased calcium entry slope, from 65% to 75% of the control (*p*<0.05, one-way ANOVA). LukS-PV Y191A was the only mutant to display a significantly increased ability to activate neutrophils, with a calcium entry slope of 135% of the control (*p*<0.01, one-way ANOVA).

### Pore-forming capacity of LukS-PV mutants

**Figure 4:**
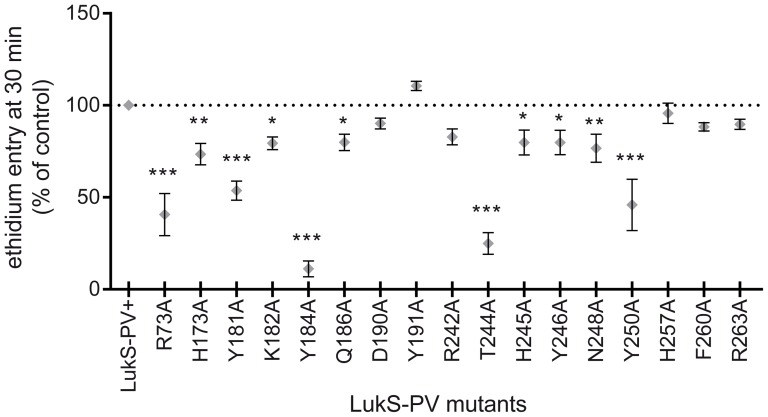
Pore-forming activity of LukS-PV and corresponding mutants. Flow cytometry analysis of ethidium entry in neutrophils 30-PV (wild type or mutant, 0.02 nM) in combination with an excess of LukF-PV (10 nM). Neutrophils were incubated with 4 μM ethidium bromide prior to toxin addition. Values of ethidium entry are expressed as percent of the control (wild type LukS-PV) activity. Statistical analysis: ns, non-significant; *, *p*<0.05; **, *p*<0.01; ***, *p*<0.001 (one-way ANOVA with Dunnett's post test, *n* = 3).

The pore-forming ability of the LukS-PV mutants was investigated by the measurement of ethidium entry through the pore using flow cytometry. All tested mutants formed pores, most with a slightly decreased ability (Fig. 4 and Table 1). Four mutants, R73A, Y184A, T244A and Y250A, had a significantly affected pore-forming activity, with at least a 50% reduction in ethidium bromide entry at 30 min. All other mutants had a pore forming activity close to that observed with wild-type LukS-PV. Interestingly, the LukS-PV Y191A mutant, which already exhibited increased hPMN activation, also displayed a slightly increased pore-forming capacity (111%) compared to LukS-PV.

### Structure determination of LukS-PV mutants

Six of the seventeen studied mutants were subjected to structural analysis: Y184A, T244A, H245A, Y246A, N248A, and Y250A. Crystals were obtained for all of the above but those obtained for the H245A LukS-PV mutant diffracted very poorly and were systematically split; therefore, the structure of this mutant could not be solved.

The T244A, Y246A and N248A mutated proteins crystallized in identical conditions (50% PEG 200, 0.1 M MES-NaOH, pH 7.50). The corresponding crystals belonged to the *P*4_3_2_1_2 space group, with similar cell parameters (a = b∼94 Å and c∼308 Å) and 4 molecules per asymmetric unit. These crystals diffracted X-rays at medium resolution (2.50 to 2.80 Å). Crystals of the Y184A mutant were obtained at a lower pH 6.50, but the conditions were otherwise identical. The latter belonged to the *P*4_1_2_1_2 space group with cell parameters a = 104.86 Å and c = 106.87 Å, 1 molecule per asymmetric unit, and diffracted up to 2.33 Å. The Y250A mutant crystallized in 5% PEG 6000, 0.1 M sodium citrate, pH 4.0. Crystals were orthorhombic, space group *P*2_1_2_1_2, with cell parameters a = 85.30 Å, b = 84.29 Å and c = 38.09 Å and 1 molecule per asymmetric unit. Crystals diffracted up to 1.55 Å. Since no crystal was isomorphous to the crystals of wild-type LukS-PV [35], molecular replacement was performed in all cases. This allowed the identification of a clear and unique solution. Refinement statistics are provided in Table 2.

**Table 2:** Crystallographic data and refinement statistics of the LukS-PV mutants^1^.

	Y184A	T244A	Y246A	N248A	Y250A
Crystallization condition	40% PEG 200 NaMES 0.1 M pH 6.50	40% PEG 200 NaMES 0.1 M pH 7.50	40% PEG 200 NaMES 0.1 M pH 7.50	40% PEG 200 NaMES 0.1 M pH 7.50	5% PEG 6000 NaCitrate 0.1 M pH 4.00
Data collection and processing					
Beamline	ID29	ID23-EH2	ID14-EH1	ID23-EH2	ID14-EH4
Spacegroup	P4_1_2_1_2	P4_3_2_1_2	P4_3_2_1_2	P4_3_2_1_2	P2_1_2_1_2
Cell parameters (Å)	a = 104.87 c = 106.89	a = 94.47 c = 310.31	a = 94.19 c = 306.44	a = 93.99 c = 309.39	a = 85.30 b = 89.29 c = 38.09
Resolution limits (Å)	47.60–2.20 (2.30–2.20)	46.70–2.75 (2.80–2.75)	47.05–2.50 (2.64–2.50)	46.99–2.80 (2.90–2.80)	30.84–1.55 (1.60–1.55)
Nb. of observations	222,233 (27,688)	231,080 (4,712)	213,925 (28,588)	291,382 (8,514)	299,073 (22,054)
Nb. of unique reflection	30,803 (3,759)	36,657 (1,606)	46,924 (6,622)	34,936 (3,263)	43,049 (3,855)
Multiplicity	7.2 (7.4)	6.3 (2.9)	4.6 (4.3)	8.3 (2.6)	6.9 (3.6)
Completeness	99.8 (99.7)	97.3 (83.0)	96.9 (95.7)	99.1 (94.6)	99.7 (99.9)
*R*_sym_	0.070 (0.915)	0.110 (0.834)	0.149 (0.827)	0.122 (0.649)	0.067 (0.753)
I/σ	16.2 (2.7)	15.1 (2.0)	10.0 (1.8)	16.0 (1.9)	18.8 (3.2)
Refinement					
Resolution limits (Å)	47.60–2.20	46.70–2.75	47.05–2.50	46.99–2.80	30.84–1.55
Nb. of reflections	30,803	36,656	46,908	34,936	43,037
*R*_factor_	0.210	0.198	0.185	0.224	0.167
*R*_free_	0.218	0.246	0.228	0.245	0.189
Nb. of molecules/A.U.	1	4	4	4	1
Nb. of atoms					
Protein	2,171	8,804	8,761	8,719	2,242
Water	106	319	473	96	297
Others	12	none	none	none	30
R.m.s. deviations					
Bond lengths (Å)	0.010	0.010	0.010	0.008	0.009
Bond angles (°)	1.12	1.21	1.17	1.09	1.06
Peptide ω angles (°)	4.14	3.34	3.69	2.79	4.37
PDB ID	4IYT	4IYC	4J0O	4IZL	4IYA

1 Numbers in parentheses report statistics for the highest resolution shell.

**Figure 5:**
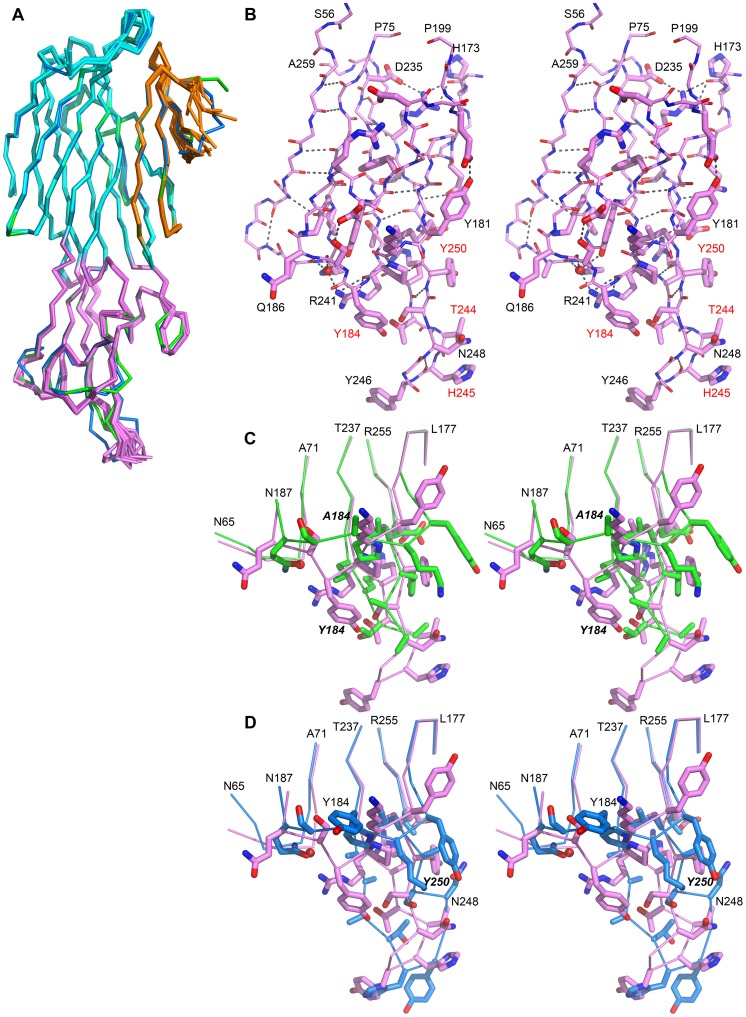
Structural variations of LukS-PV upon mutations in the rim domain. A. Superposition of the Cα trace of all available structures of LukS-PV proteins, wild-type or mutants. The Y184A mutant structure is represented in green and the Y250A mutant structure in blue. For the other structures, the three structural domains are highlighted: β-sandwich (cyan), stem (orange) and rim (purple). B. Close-up stereo view of the rim domain of the LukS-PV wild-type structure, shown as stick (thin for main-chain and thick for side-chains). Residues labeled in red correspond to the most sensitive position as identified in this work. C. Stereo view of portion of the rim domain of wild-type LukS-PV (purple) and of the Y184A mutant structure (green). D. Stereo view of portion of the rim domain of wild-type LukS-PV (purple) and of the Y250A mutant structure (blue). Panels B to D, hydrogen bonds are represented as grey dots, the orientation of the image is the same as in A. Panels C and D, mutated positions are labeled in italic.

The overall three-dimensional structures of the mutants were very similar to the wild-type structure. Indeed, all secondary structure elements were preserved and local differences were only observed in certain loops (Fig. 5A). The *β-sandwich* domain was the most conserved, with r.m.s. deviations after superposition of equivalent Cα atoms below 0.63 Å. The *stem* domain appeared more flexible with r.m.s. deviations ranging from 0.21 Å to 2.21 Å.

In the case of the *rim* domain, wild-type LukS-PV and mutants T244A, Y246A and N248A displayed only small differences, with r.m.s. deviations comprised between 0.20 Å and 0.30 Å, whereas the structure of the rim domain of the Y184A and Y250A mutants were markedly different, with r.m.s. deviations of 1.10 Å and 1.28 Å, respectively, when compared to the wild-type LukS-PV structure (Fig. 5). These differences were mostly concentrated in the V169–Q186 and T244–N248 loops, with mutants Y184A and Y250A being the most divergent. Of particular note, the *rim* region was generally associated with weak electron density, and could not be completely built in most structures, except in the case of the Y250A mutant.

## Discussion

Specific binding of LukS-PV to the surface of human neutrophils has been shown to require the presence of a functional C5a receptor [41], and occurs with an apparent affinity varying between 0.06 to 6 nM depending on the protein purification tag and the methodology used [31], [40], [41]. Non-specific binding to cellular or artificial membranes has also been shown to occur, however only when using micromolar concentrations of LukS-PV [48]. All of the experiments described in the present study were performed at nanomolar concentrations of wild-type or mutant LukS-PV, ensuring that only specific binding to the surface of neutrophils, *i.e.* to the C5a receptor, could occur. This was confirmed through the use of undifferentiated U937 cells that do not naturally express C5aR and do not significantly bind LukS-PV* even at protein concentrations up to 500 nM, whereas this binding was observed with a *K_d_* of 320 pM with U937 cells expressing human C5aR.

Among the seventeen LukS-PV mutants investigated, eight mutants (H173A, Q186A, D190A, Y191A, R242A, H257A, F260A and R263A) displayed only marginally altered biological properties. Five mutants (Y181A, K182A, H245A, Y246A and N248A) were partially affected. Four LukS-PV mutants (R73A, Y184A, T244A, and Y250) displayed significant alterations for all measured parameters, *i.e.* at least 50-fold decrease in affinity for hPMN membranes, 2-fold decrease in the slope of Ca^2+^ influx and 2- to 10-fold reduction in pore-forming capacity. The most dramatically reduced affinity of the LukS-PV mutants for the hPMN membrane led to a reduced amount of bound leukotoxin, which resulted in a decreased number of both activated hPMNs and functional pores. However, none of the studied mutants was completely inactive. Indeed, the *K_i_* of wild-type LukS-PV was 0.03 nM and even mutants displaying a 200-fold degradation in binding still displayed a *K_i_* of about 10 nM, resulting in a dramatically reduced but still recordable biological activity. Comparable variations of *K_i_* were observed when LukS-PV mutants were tested for binding to recombinant undifferentiated U937-C5aR (Figure 2). The absence of measurable binding to undifferentiated U937 cells indicates that the presence of the C5a receptor is required for the initial binding of LukS-PV and for subsequent events of toxin formation.

The *rim* domain is known to be responsible for the interaction of leukotoxins with target cell membranes while the structure of the γ-hemolysin pore [7] indicates that the loops extending at the bottom of the *rim* domain are likely involved in this interaction. In LukS-PV, this area would correspond to residues 181–186 and 241–251. Three of the four most sensitive residues (Y184, T244 and Y250) are found in this region, at close distance from each other (Fig. 1C). Residue R73, the fourth most affected residue, is located close to the β-sandwich domain (Fig. 1C). In close proximity to residues Y184, T244 and Y250, residues R242, H245 and Y246 also induced altered biological activities when mutated to alanine, albeit to a somewhat lesser extent. Binding affinities of LukS-PV Y184, T244 and Y250 mutants underwent a 14- to 61-fold decrease with respect to the value obtained for the wild-type protein. These results hence identify the bottom loop 240–250 as well as residue Y184 as being crucial for LukS-PV binding to the target cell membrane. Residues Y184, T244, Y250, R242, H245, and Y246 are located on poorly conserved regions among leukotoxins, except between LukS-PV and HlgC. Residues R73, H245, Y246 and N248 are specific to the two proteins, whereas Y184 of LukS-PV is replaced by a histidine residue in HlgC (Figure 1A). In these two proteins, a four-residue insertion is found in the loop T240–Y250 compared to other class S components. This loop has furthermore been shown to be essential for γ-hemolysin activity [49] which has a different cell-target spectrum, although its direct role in LukS-PV and HlgC activity has never been demonstrated until now. Indeed, our study provides clear evidence that the corresponding segment in LukS-PV is crucial for its binding to the C5a receptor. In this region, the only differences in sequence between Luks-PV and HlgC are located at positions 240 (Thr in LukS-PV and Ile in HlgC), 241 (Arg in LukS-PV and Lys in HlgC) and 243 (Thr in LukS-PV and Ser in HlgC). This T240-T243 stretch is strictly conserved among 40 known variants of LukS-PV. Such differences between LukS-PV, HlgC and other class S components may contribute to differences in their target cell spectrum as well as the binding capacities of PVL and HlgC/HlgB.

The three dimensional structures of five of the seventeen investigated LukS-PV mutants were obtained: Y184A, T244A, Y246A, N248A, and Y250A. This provided a unique opportunity to correlate their altered biological behavior to structural properties. In order to sample all possible conformations of LukS-PV, all structures, including the multiple structures resulting from non-crystallographic symmetry, were superimposed onto the A-chain of the wild-type LukS-PV structure (PDB entry 1T5R). With the 13 structures provided by this study, and since there are 8 molecules in the asymmetric unit of the wild-type crystals, 21 structures could be compared (Fig. 5A). The comparison clearly enabled to identify regions with the highest structural flexibility: the *stem* region and the three loops of the *rim* domain. In the structure of the soluble form of both S and F leukotoxin components, the *stem* region is comprised of a 3-stranded antiparallel β-sheet and a connecting loop (residues P121–F129 in LukS-PV), closely apposed against the *β-sandwich* domain. Our comparison of all available structures of the LukS-PV proteins indicates that this connecting loop is highly flexible. In all structures, except for the Y250A mutant, electron density was very weak in this region and the loop could not be built completely. This structural flexibility is not surprising since the *stem* region has to undergo major structural rearrangement when it deploys into the membrane and contributes two β-strands to the β-barrel pore.

The *rim* domain is built from three stretches of residues: S56–P75, A160–H203, and N233–H257 [35]. Both S56–P75 and N233–H257 segments are comprised of β-strands that extend from/into the *β-sandwich* domain. They form a 4-stranded β-sheet onto which residues A160–H203 are apposed. This latter stretch of residues includes a small 2-stranded β-sheet (N161–T165 and G168–G172) and a long loop (H173–H203) that includes three short helical segments (P188–Y191, D195–E197, and P200–H203). The conformation of this loop is stabilized by several intra-loop hydrogen bonds, whereas only a few interactions with the other regions of the *rim* domain were found (Fig. 5B). In most structures of LukS-PV, the *rim* domain conformation is well conserved, with the exception of the R241–A250 loop where certain local variations were observed (Fig. 5A). Other significant variations in the conformation of the *rim* domain were only found in the structure of the Y184A mutant, where residues V179–Q186 were affected (Fig. 5C), as well as in the conformation of the Y250A mutant, where two loops (V179–Q186 and R241–A250) showed structural variations (Fig. 5D). For both Y184A and Y250A mutants, crystallization was achieved in different conditions than those for wild-type LukS-PV and the other mutants, resulting in a different crystal packing. This alone suggests that the structures of mutants Y184A and Y250A differ from the others, preventing crystallization in similar conditions. As the result of the Y184A mutation, the hydrogen bond found in the wild-type LukS-PV structure between Y184OH and S249OG was lost, and the loop was reorganized with a displacement of 6.0 Å for the CA atom at position 184 (Fig. 5C). In the Y250A mutant structure, the removal of the tyrosine side chain allowed the displacement of the N248 side chain, which in turn drove the reorganization of residues R241–Y250. Space was therefore provided for the displacement of the V179–Q186 loop, which adopted a conformation close to that observed in the case of the Y184A mutant (Fig. 5D).

**Figure 6:**
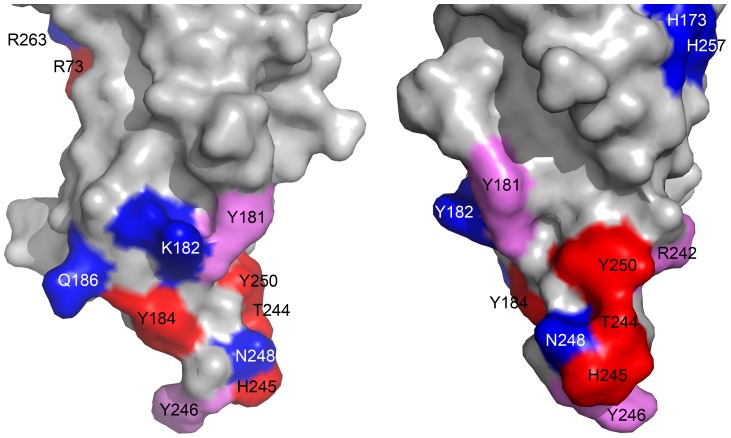
Molecular surface of the rim domain of LukS-PV. Residues identified in this study as important for the binding of LukS-PV on the C5a receptor (*K_i_* increased more than 50 fold upon mutation to Ala) are depicted in red. Mutated residues affecting binding to a lesser extent (i.e. increase in *K_i_* by a factor between 5 and 50) are depicted in pink whereas residues for which no effect on binding was found upon mutation (increase in *K_i_* less than 3 fold) are depicted in blue (Table 1). Two orthogonal views around a vertical axis are presented, with the orientation on the left being the same as in figure 5.

The structural variations observed in this study suggest that the *rim* domain of LukS-PV may have the ability to easily adapt its conformation in order to bind to the C5a receptor present in the target cell membrane. Our results show that the *rim* domain of LukS-PV likely displays the required plasticity for these events, and that tyrosine residues 184 and 250 may be of paramount importance in this process, since the corresponding mutants are among the most affected. Moreover, the functional studies performed herein indicate that loop 240–250 is crucial for the binding of LukS-PV to C5aR. Indeed, three of the five most sensitive residues identified (T244, H245 and Y250) are located in this loop and form a surface that could represent the principal binding site for the receptor (Figure 6). The fourth most affected residue, Y184, also interacts with this loop. To date, no natural mutation of LukS-PV has been reported that would affect its specific binding to the membrane receptor. Recent results obtained with LukE have identified regions Q180–A193 and L234–R268 as essential for LukE/LukD cytotoxicity, but only region Q180–A193 as being required for binding to CXCR1 and CXCR2 [43], whereas in the case of LukH (LukA), residue 323 was found to be crucial for binding to CD11b [50] This suggests that the mechanism of receptor recognition may not be unique among all leukotoxins. Moreover, binding of the S component of leukotoxins to its cognate receptor is not sufficient in itself to result in pore formation, which requires the recruitment of the F component and the formation of the adequate hetero-oligomer in order to form the prepore and, eventually, the pore. All of these steps, which potentially require specific and major interactions of LukS-PV with the extracellular region of C5aR, remain to be characterized at the molecular level. Such data along with a better understanding of these critical steps involved in LukS-PV binding are of crucial importance for pharmacological purposes.

## Experimental Procedures

### Bacterial strains and vectors

*Escherichia coli* XL1 Blue cells [*recA1 endA1 gyrA96 thi1 hsdR17 supE44 relA1 lac* (F′ *proAB lacIqZΔM15 Tn10* (*tet* ^r^))] (Stratagene, Agilent Technologies, Massy, France) were used as recipient cells for transformation with recombinant pGEX-6P-1∼Panton-Valentine leukocidin genes (Genbank:X72700) (GE Healthcare Life Science, France, [5]). *E. coli* BL21 [F^−^, *ompT, hsdS* (*rB* ^−^, *mB* ^−^), *gal*] was used for over-expression of the glutathione-S-transferase (GST)∼leukotoxin fusion genes, according to the manufacturer's instructions (GE-Healthcare).

### Alanine scanning site-directed mutagenesis

LukS-PV mutants were constructed by means of the QuickChange mutagenesis protocol (Stratagene) using Phusion Hot Start DNA polymerase (Finnzyme, Espoo, Finland) and dedicated oligonucleotides as previously described [5]. All mutated genes were verified by DNA sequencing.

### Expression and purification of leukotoxins

Wild-type and mutant leukotoxins were expressed and purified as previously described [30]. Briefly, recombinant BL21 *E. coli* cells were grown in 2×TY medium (bacto-tryptone 17 g/l, bacto-yeast extract 10 g/l, NaCl 5 g/l), and protein expression was induced with 0.2 mM IPTG. GST-fusion proteins were purified by affinity chromatography on glutathione-Sepharose 4B (GE Healthcare), followed by a SP-sepharose cation-exchange run on a Fast Protein Liquid Chromatography AKTAPurifyer, after removal of the glutathione S-transferase tag with PreScission protease (GE Healthcare). The identity and purity of proteins were confirmed by radial gel immunoprecipitation against native antigens (0.6% (w/v) agarose in phosphate buffered saline) and 10–15% (w/v) SDS-PAGE. Proteins were stored at −80°C.

### hPMN purification and activation

Blood samples of anonymous healthy volunteer donors were purchased at the “Etablissement Français du Sang, Strasbourg, France” in accordance with convention n°SG-CLI-003. Human polymorphonuclear neutrophil leukocytes (neutrophils) were purified from buffy coats as previously described [47], and suspended at 5×10^5^ cells/ml (unless specified otherwise) in 10 mM HEPES, 140 mM NaCl, 5 mM KCl, 10 mM glucose, 0.1 mM EGTA pH 7.3. Human PMN activation was monitored by following the variation in intracellular free Ca^2+^. Calcium changes were determined by recording the variations in emitted fluorescence of Fluo3-loaded neutrophils as previously described [31]. Briefly, neutrophils were incubated with 2 μM Fluo3-AM^®^ (Molecular Probes, Eugene, USA) for 45 min at room temperature, then washed and resuspended twice in HEPES buffer. Five minutes prior to toxin addition, 1.1 mM CaCl_2_ was added to hPMN suspensions (6×10^6^ cells/ml). Variations in fluorescence intensity of Fluo3 were recorded with a spectrofluorometer (Deltascan, PTI, USA) at λ_Ex_ = 488 nm and λ_Em_ = 530 nm.

### Undifferentiated U937 and U937-C5aR cell cultures

Undifferentiated U937 and U937-C5aR cells, which respectively do not express or stably express the C5aR receptor [51], were a generous gift from Pr. J.A. van Strijp (Utrecht University, The Netherlands). Cells were cultured as 50 ml suspension at 37°C under a 5% CO_2_ atmosphere in 250 ml flasks in RPMI-1640 medium supplemented with 10% (v/v) of decomplemented fetal calf serum (Life Technologies, Carlsbad, USA) and 0.1% (w/v) of both penicillin and streptomycin (InVitrogen, Paisley, UK).

### Pore formation measurements

Pore formation was revealed by the penetration of ethidium into cells [52]. Neutrophils (5×10^5^ cells/ml) were pre-incubated for 10 min with 4 μM ethidium bromide. Measurements were initiated directly after the simultaneous addition of 0.1 nM of the S component and 10 nM of LukF-PV. Ethidium fluorescence of 3×10^3^ hPMNs was measured using a FacSort flow cytometer (Becton-Dickinson, Le Pont de Claix, France) equipped with a 15-mW, 488-nm, argon-ion laser. Data were acquired using CellQuest Pro software (Becton-Dickinson). Ethidium fluorescence of hPMNs after a 30-min incubation with PVL was calculated and normalized with respect to the wild-type LukS-PV control. The results for 3 different donors were averaged and expressed as percentages of fluorescence values recorded with dead cells. Baseline values were obtained for each series of data from a control without addition of toxin, and were systematically subtracted from the results of other assays.

### Leukotoxin binding assays

#### K_d_ determination

The dissociation constants, *K_d_*, were determined using a binding saturation experiment with increasing concentrations of fluorescein-labeled LukS-PV* (0.01 nM to 10 nM for hPMNs; 0.1 nM to 500 nM for U937 cells), as previously described [31]. The amount of labeled protein bound to the cell surface was measured by flow cytometry as cell fluorescence at λ_Em_ = 530 nm and expressed as the percentage of maximum fluorescence obtained at the highest concentration of LukS-PV*. Experimental data were fitted using GraphPad Prism version 5.04 for Windows (GraphPad Software, San Diego, USA). *K_d_* values were calculated by a non-linear regression using the “One site - Specific binding” equation.

#### K_i_ determination

Fluorescein-labeled LukS-PV* (1 nM) was displaced by increasing concentrations of various non-labeled LukS-PV or mutants (0.03 nM to 500 nM). The amount of LukS-PV* bound to the cell surface was measured by flow cytometry as the amount of cell fluorescence at λ_Em_ = 530 nm. Fifty percent effective concentrations (EC_50_) were calculated by GraphPad Prism using the non-linear regression “one site binding” equation. The equation of Cheng and Prusoff [53] was used to calculate the inhibition constant, *K_i_*, from the EC_50_ value (parameters: *K_d_* LukS-PV* = 0.066 nM; [LukS-PV*] = 1 nM). 
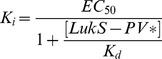



### Protein crystallization and structure determination

Proteins were conditioned in 50 mM MES-NaOH buffer, 50 mM NaCl, pH 6.5 at approximately 10 mg/ml. Crystallization trials were implemented using the sitting drop method and a NanoDrop ExtY automated crystallization platform (Innovadyne) at 285 K. Drops were generated by mixing 200 nL of protein solution to the same volume of crystallization solution (Table 2).

Crystals were cryo-protected by brief immersion in the crystallization solution supplemented with 20% (v/v) ethylene glycol before being transferred into a gaseous nitrogen flux at 100 K. All data collections were performed at the European Radiation Synchrotron Facility (ESRF, Grenoble, France). Data processing was initially performed using autoPROC [54], and optimized with XDS [55] and SCALA [56] (Table 2). All subsequent operations were performed using the CCP4 program suite [57].

Structures were solved using the molecular replacement method, using Phaser 2.3 [58], with the structure of the wild-type LukS-PV as starting model [35]. Refinement was performed with Buster (GlobalPhasing, UK) and Coot softwares [59] (Table 2).
